# Managing fatigue with methylphenidate and physical activity during cancer immunotherapy treatment

**DOI:** 10.1017/S1478951525101375

**Published:** 2025-12-22

**Authors:** Sriram Yennurajalingam, Bryan Fellman, Lisa Williams, Karen Basen-Engquiest, Eduardo Bruera

**Affiliations:** 1 Department of Palliative Care, Rehabilitation, and Integrative Medicine, Division of Cancer Medicine, University of Texas MD Anderson Cancer Center, Houston, TX, USA; 2 Department of Biostatistics, The University of Texas MD Anderson Cancer Center, Houston, TX, USA; 3 Department of Health Disparities Research, The University of Texas MD Anderson Cancer Center, Houston, TX, USA

**Keywords:** Palliative care, fatigue, methylphenidate, physical activity, supportive care

## Abstract

**Objectives:**

Despite the high frequency and severity of fatigue among patients with advanced cancer receiving immunotherapy, there are limited treatment options available. The aim of the study was to explore the effects of the methylphenidate (MP) with standardized physical activity (PA) on cancer related fatigue (CRF).

**Methods:**

In this pilot study, patients with advanced cancer with clinically significant CRF (<34 on Functional Assessment of Cancer Illness Therapy – fatigue scale, FACIT-F), on anti-PD1 immunotherapy were eligible. Patients were randomized to standardized PA with either patient-controlled MP 5 mg (MP + PA arm) or matching Placebo (Pl + PA arm) twice daily for 14 days. The primary outcome was the change in the FACIT-F score. Secondary outcomes included changes in fatigue dimensions (Multidimensional Fatigue Symptom Inventory-Short Form (MSFI-SF), Functional Assessment of Cancer Therapy – General (FACT-G), Patient-Reported Outcome Measurement Information System-Fatigue (PROMIS-F), and hospital anxiety and Depression Scale (HADS).

**Results:**

Of the 40 randomized patients, 34 were evaluable. The FACIT-F scores significantly improved in both the arms with mean (SD) change, effect size (ES) of 11(14), 0.87(*P <* .001); and 9(12), 0.74(*P =* .04) in MP + PA, and Pl + PA arms respectively. We also found significant improvements in PROMIS-F, ES − 1.05(*P =* .003), MFSI-SF(global), ES − 1.32(*P <* .001), and HADS-depression, ES − 0.92(*P =* .004) in the MP + PA arm; There were no significant differences in adverse events between groups.

**Significance of results:**

Our preliminary study found MP + PA was associated with significant improvement in CRF scores. The fatigue dimensions and depression scores significantly improved in the MP + PA arm. Further comparative studies using MP + PA for CRF are justified.

## Introduction

Cancer-related fatigue (CRF) in patients with advanced cancer is more frequent and severe than in those with early cancer or in cancer survivors (Bower 2014). In patients with advanced cancer, moderate to severe fatigue is associated with poor quality of life outcomes, performance status scores, frailty, and lower overall survival (Stone et al. 2000; Lawrence et al. 2004; Hofman et al. 2007; Minasian et al. 2007; Pollack 2009; Bower 2014; Charalambous and Kouta 2016; Dittus et al. 2017). The National Comprehensive Cancer Network defines CRF as a “distressing, persistent, subjective sense of physical, emotional, and/or cognitive tiredness or exhaustion related to cancer or cancer treatment that is not proportional to activity and that interferes with usual functioning”(Berger et al. 2015).

Checkpoint inhibitors like anti-PD-1 inhibitors have transformed outcomes in patients with advanced cancer (Gotwals et al. 2017; Johnson et al. 2022; Sun et al. 2023; Benelli et al. 2024). However, up to 40% of these patients report CRF during or after treatment (Abdel-Rahman et al. 2016; Postow et al. 2018; Zhou et al. 2021; Kiss et al. 2022). CRF in this population may be due to its various contributing dimensions, including mood, central nervous system (CNS), physical functioning, immune activation, cytokine dysregulation, and chronic inflammation (Bower 2014; Abdel-Rahman et al. 2016; Azeem Khan et al. 2021). CRF in this population is often underrecognized and undertreated, potentially affecting treatment adherence and clinical outcomes. However, there are few evidence-based interventions specifically tailored to immunotherapy-related CRF (Azeem Khan et al. 2021; Bower et al. 2024). Established therapies for CRF, such as physical activity (PA) in patients with advanced cancer, suggest only low to modest efficacy (Dittus et al. 2017; Peddle‐McIntyre et al. 2017; Nadler et al. 2019; Fabi et al. 2020; Ligibel et al. 2022; Tanriverdi et al. 2023; Toohey et al. 2023; Bower et al. 2024; Hiensch et al. 2024). In advanced cancer patients, results of the studies using pharmaceutical agents for CRF have been mixed (Peuckmann‐Post et al. 2010; Yennurajalingam and Bruera 2014; Fabi et al. 2020; Mochamat et al. 2021; Yennurajalingam et al. 2022b; Stone et al. 2024). Prior studies conducted by our team and others demonstrated that MP is safe and potentially efficacious in patients with advanced cancer (Bruera et al. 2003, 2006; Pedersen et al. 2020; Belloni et al. 2021). However, both our findings and those of others have shown that MP was not significantly more effective than placebo in reducing CRF (Bruera et al. 2003, 2006; Belloni et al. 2021; Centeno et al. 2022; Stone et al. 2024). This may be attributed to the multifactorial nature of fatigue in this population. It is possible that no single treatment modality will sufficiently address all causes of CRF in an individual. Recent studies suggest that the most efficient strategies may be to combine both pharmacological and nonpharmacological approaches that have shown promise in order to target the multifactorial causes of CRF in patients with advanced cancer (Barsevick et al. 2013; Chin-Yee et al. 2024).

In this study, our goal was to address the multifactorial nature of CRF in a specific subgroup of advanced cancer patients receiving anti-PD-1 immunotherapy by combining two of the most frequently studied interventions: PA and MP. We hypothesized that the improvement in CRF may be driven by a synergistic interaction, wherein MP enhances the fatigue-alleviating effects of PA. Prior research suggests that PA positively influences health-related fitness, mood, cognition, and psychological well-being – factors known to mediate CRF ( McNeely and Courneya 2010; Larkin et al. 2014; Yang and Wang 2017; Mast et al. 2025). Additionally, PA exerts immune-modulatory effects, including reductions in proinflammatory cytokines such as IL-6 and TNF-α, which are implicated in treatment-related fatigue (Greenberg et al. 1993; Segal et al. 2009; Nimmo et al. 2013; You et al. 2013; Rogers et al. 2014). MP, through its CNS activity – blocking the reuptake of dopamine and norepinephrine and acting on the reticular activating system – may improve arousal, mood, and cognitive function (Gualtieri et al. 1986; Cooper et al. 2005; Watson et al. 2005; Bart et al. 2010). These effects can complement and amplify the benefits of exercise, particularly in domains such as mood regulation and sleep quality (Gualtieri et al. 1986; Cooper et al. 2005; Watson et al. 2005; Bart et al. 2010). We postulate that MP enhances the effects of PA on CRF by increasing arousal and cognitive function, while PA may potentiate the mood and sleep benefits of MP (Schwartz et al. 2002), resulting in a synergistic improvement in CRF.

Therefore, in this pilot study, our primary aim was to explore the preliminary effects of MP with PA (MP + PA) on CRF as assessed by the change in Functional Assessment of Cancer Illness Therapy (FACIT-F) – fatigue scale between baseline and the end of 2 weeks. We also examined changes in the Multidimensional Fatigue Symptom Inventory-Short Form (MFSI-SF), Patient-Reported Outcome Measurement Information System (PROMIS)-Fatigue, Edmonton Symptom Assessment Scale (ESAS), and Functional Assessment of Cancer Therapy (FACT-G) scores and adverse events as assessed using the National Cancer Institute’s Common Terminology Criteria for Adverse Events (NCI CTCAE) version 4 after study treatments.

## Methods

The institutional review board of the University of Texas M.D. Anderson Cancer (UT MDACC) Center approved this protocol, and all participants signed an informed consent as a condition of enrollment in the trial. This study was conducted from May 2018 to August 2021.

### Participants

Patients were enrolled from the outpatient clinics of supportive care and oncology at the UT MDACC. Patients’ eligibility criteria included (a) having a diagnosis of advanced cancer and being on anti-PD1 immunotherapy, (b) presence of fatigue as defined FACIT-F subscale of ≤ 34 on a 0 to 52 scale (in which 52 = no fatigue and 0 = worst possible fatigue), (c) normal cognition, (d) life expectancy of at least 4 months, (e) no severe cardiac disease (New York Heart Association functional class III or IV), (f) not regularly participating in moderate- or vigorous-intensity PA for ≥ 30 minutes at least 5 times a week and strength training for ≥ 2 days, (g) no falls in the past 30 days, (h) not currently taking MP or have taken it within the previous 10 days, (i) have no major contraindication to MP (e.g., allergy/hypersensitivity to study medications or their constituents), (j) not on monoamine oxidase inhibitors, or clonidine, or history of glaucoma, (k) patients with CAGE–AID <2. (l) no history of tachycardia and/or uncontrolled hypertension, and (m) not currently receiving anticoagulants, anticonvulsants (phenobarbital, diphenylhydantoin, primidone), phenylbutazone, and/or tricyclic drugs (imipramine, clomipramine, or desipramine).

## Interventions

Forty patients were randomized to receive either a standardized PA intervention with patient-controlled MP (MP + PA arm) or a matching placebo (Pl + PA arm) at 2:1 randomization. The 2:1 randomization ratio was chosen to enrich the intervention arm (MP + PA) since the primary aim of the study was to assess changes in fatigue within this group. This design allowed for more robust preliminary data on the MP + PA intervention.

MP or Placebo: The patients in the MP + PA group received oral MP with a starting dose of 5 mg twice daily for 14 days. The last dose of the twice-a-day regimen was given prior to 3 pm, and the interval between doses was at least 2 hours. Patients had an option to have the dose escalated to 10 mg twice daily based on patient-reported fatigue and, if the patient did not have any grade >/ = 2 adverse events related to the study drug, such as restlessness, behavioral changes, dizziness, tachycardia, anorexia, itching, and nausea. All members of the research team (except the investigational pharmacist and statistician), as well as the patients, were blinded to the study medication assignment throughout the study.

PA Intervention: Patients in both study arms received standardized PA activity intervention. The PA intervention was the same as described in our previously published studies (Yennurajalingam et al. 2022a, 2021, 2025). The weekly regimen included a graded resistance exercise program and a walking regimen. We used resistance tubes as our mode of resistance. These tubes are color-coded to indicate their specific resistance level: light, moderate, or hard. The resistance exercise sessions were completed 3 days a week, allowing at least 48 hours between each session. The individual also engaged in a walking program. Since the level of aerobic fitness varied among participants, the intensity and duration of the walking program were established based on the initial evaluation of the participants’ current aerobic fitness level, assessed using the 6-minute walk test. The participants were asked to walk a minimum of 5 days a week. At the first study visit, the research coordinator met with each participant to evaluate his or her current strength and aerobic fitness level and supervised the assigned exercises. Patients then received weekly phone calls from the research nurse to assess their progress and to help them identify and overcome any barriers to completing the exercise program.

While PA interventions in cancer populations often span 4–12 weeks and include objective measures such as fitness, strength, or functional capacity (e.g., 6-minute walk test, dynamometry), the current study was designed with a different focus. The MP + PA intervention aimed to assess symptomatic improvement, specifically in CRF, rather than changes in physical performance. Given the advanced stage of disease in our patient population, longer intervention windows risk capturing deterioration due to disease progression, which could confound fatigue outcomes. Therefore, a two-week intervention period was selected to minimize this confounding and better isolate the effect of the intervention on fatigue. This shorter timeframe is particularly appropriate for patients with metastatic cancer, where clinical status can decline rapidly.

Adverse events were assessed using adverse event monitoring, and this was assessed in accordance with the NCI CTCAE version 4.

## Outcome measures

Patients’ demographic data, including age, sex, ethnicity, and cancer diagnosis, were recorded at the time of randomization.

A research coordinator supervised the patients’ completion of the FACT-G, FACIT-F, ESAS, PROMIS-Fatigue, MFSI, and HADS assessment tools.

**FACIT-F** is a validated instrument designed to assess fatigue in patients with chronic illnesses, particularly cancer (Cella et al. 1993, 2002; Butt et al. 2013). It consists of 13 items, each rated by the patient on a 5-point Likert scale ranging from 0 (“Not at all”) to 4 (“Very much”). The summed score ranges from 0 to 52, with higher scores indicating less fatigue and lower scores reflecting greater fatigue severity. A total score of 34 or below is considered indicative of clinically significant fatigue, a threshold supported by empirical validation (Yellen et al. 1997). The FACIT-F demonstrates high internal consistency (Cronbach’s α = 0.93–0.95), sensitivity of 0.92, and specificity of 0.69 (Cella et al. 2002, 1993).

**FACT-G** is a well-validated quality-of-life instrument widely used for the assessment of quality of life in clinical trials. It consists of 27 general quality-of-life questions divided into four domains (physical, social, emotional, and functional).

**ESAS** is a valid 0–10 scale used to assess 10 symptoms commonly experienced by cancer patients during the previous 24 hours: pain, fatigue, nausea, depression, anxiety, drowsiness, dyspnea, anorexia, sleep, and feelings of well-being (35, 40–42).

**PROMIS-Fatigue short form** was used to measure both the experience of fatigue and the interference of fatigue on patients’ daily activities over the past week. PROMIS-F consists of seven items with response options on a 5-point Likert scale, ranging from 1 = never to 5 = always (36, 37).

**MFSI-SF** is a 30-item scale used to assess the multidimensional nature of fatigue (Stein et al. 2004). Responses are made using a five-point scale, ranging from 0 = not at all to 4 = extremely. The MFSI-SF total scale and subscales are validated with a Cronbach α of 0.83–0.92 (Donovan et al. 2015).

The primary outcome of this study was the FACIT-F fatigue scale, a validated tool for CRF that our team and others have previously used in multiple fatigue treatment trials (Bruera et al. 2003, 2006, 2007; Minton et al. 2008; Yennurajalingam et al. 2012). In addition, our goal in this study was to determine the sensitivity to change to other CRF assessment tools such as MFSI-SF, PROMIS-fatigue, and ESAS–fatigue item that might be of interest for future CRF research.

**HADS**: Depression and anxiety symptoms were assessed by using the 14-item HADS questionnaire, which asks patients to underline the statement that most closely matches how they have been feeling in the previous week. This questionnaire has been found to be valid and reliable in clinical situations and has been widely used in medically ill patients (Johnston et al. 2000).

## Ethics approval

The University of Texas MD Anderson Cancer Center Institutional Review Board approved the protocol in accordance with the Declaration of Helsinki, and all patients were provided written informed consent for participation in the study.

## Data analysis

Descriptive statistics were summarized using medians and interquartile ranges for continuous variables, and frequencies with percentages for categorical variables. The primary objective of this pilot study was to explore the preliminary effects of MP with PA (MP + PA) on CRF as assessed by the change in Functional Assessment of Cancer Illness Therapy (FACIT-F) – fatigue scale between baseline and the end of 2 weeks. This pilot study was designed to examine the within-group effects of the combined intervention of MP + PA on CRF. It was not powered to detect between-group differences due to its exploratory nature.

Sample size calculations were based on prior research (50), assuming a robust response rate of 0.33, the estimated 95% confidence interval half-widths are approximately 0.14 for N = 47 (CI: 0.20–0.48) and 0.20 for N = 23 (CI: 0.15–0.56) in the MP + PA and Pl + PA arms, respectively, with a total sample size of 70.

Primary (FACIT-F) and secondary outcomes (MFSI-SF, PROMIS, HADS, FACT-G, ESAS) were analyzed to evaluate treatment effects from baseline to the primary endpoint (end of week 2) using t-tests or rank-sum tests, depending on data distribution.

## Results

**Figure 1.**
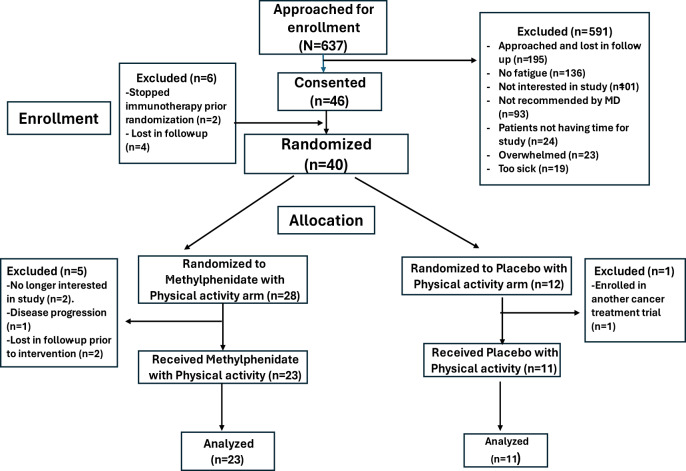
Consort diagram.

Figure 1 shows the details of patient enrollment, allocation, and analysis. 34 of the 40 randomized patients were evaluable. Reasons for the patients not receiving study interventions after randomization include: (a) withdrew consent as the patient decided to participate in a cancer treatment clinical trial (*n* = 1), (b) Withdrew from study due to disease progression (*n* = 2), (c) Patient was no longer interested in the study (*n* = 1), and (d) lost in follow-up (*n* = 2).

Table 1 shows the demographic and baseline clinical characteristics. 35% were female, the median age was 66 years, and the baseline fatigue severity (FACIT-F) score was 20. Adherence rates for study medication were 87% in the MP + PA arm, and 100% in the Pl + PA arm (*P =* .54). The median (IQR) number of pills used was 28 (28, 28), and the median MP dose was 10 mg of MP (2 pills) per day.

**Table 1.** Demographics and clinical characteristics

Characteristics, *N* (%)	MP + PA arm (*n* = 28)	Pl + PA arm (*n* = 12)	Total (*N* = 40)	*P*^a^
Age, years, median (IQR)	67(61, 71)	65(53, 74)	66(60, 71)	.87
Sex				.40
Female	11(39.3)	3(25.0)	14(35.0)	
Race				1.00
American Indian or Alaska Native	1(3.6)	0(0.0)	1(2.5)	
Black or African American	1(3.6)	0(0.0)	1(2.5)	
White or Caucasian	26(92.8)	12(100.0)	38(95.0)	
Marital status				.63
Divorced	4(14.3)	2(16.7)	6(15)	
Married	23(82.1)	10(83.3)	33(82.5)	
Widowed	1(3.6)	0(0.0)	1(2.5)	
Education level, years, median (IQR)	16(14, 17)	16(15, 17)	16(14, 17)	.58
Cancer diagnosis				.06
Gastrointestinal	1(3.6)	0(0.0)	1(2.5)	
Genitourinary	8(28.6)	2(16.7)	10(25.0)	
Head and neck	1(3.6)	0(0.0)	1(2.5)	
Melanoma	12(42.9)	9(75.0)	21(52.5)	
Thoracic	6(21.4)	1(8.3)	7(17.5)	
Subjective measures, median (IQR)	**MP + PA arm (*n* = 26)**	**Pl + PA arm (*n* = 12)**	**Total (*N* = 38)**	***P***
FACIT fatigue	19(12, 26)	20(16, 25)	20(14, 26)	.57
PROMISE T-score				
Fatigue	61(55, 65)	61(57, 65)	61(55, 65)	.73
MFSI-SF				
Global score	32(17, 43)	29(20, 48)	31(20, 43)	.73
General fatigue	18(12, 21)	18(17, 23)	18(13, 22)	.22
Physical fatigue	11(5, 13)	9(7, 10)	9(7, 13)	.64
Emotional fatigue	10(2, 13)	6(3, 11)	8(2, 12)	.63
Mental fatigue	7(2, 12)	9(3, 15)	8(2, 12)	.54
Vigor	13(10, 16)	13(11, 16)	13(10, 16)	.92
FACT-G total score	64(60, 70)	57(56, 64)	62(57, 68)	.06
Physical well-being	12(6, 17)	9(5, 11)	10(5, 16)	.14
Social/family well-being	26(23, 28)	27(24, 28)	26(23, 28)	.68
Emotional well-being	8(5, 11)	6(4, 10)	8(5, 10)	.36
Functional well-being	19(16, 24)	18(17, 23)	19(16, 24)	.90
HADS				
Anxiety	5(1, 8)	4(3, 7)	5(2, 8)	.96
Depression	7(4, 9)	6(5, 7)	6(5, 9)	.56
ESAS				
Fatigue	6(5, 8)	7(5, 8)	6(5, 8)	.90
Pain	3(1, 6)	2(1, 3)	2(1, 5)	.06

Abbreviations: MP + PA, Methylphenidate and Physical Activity; Pl + PA, Placebo and Physical Activity; IQR, interquartile. FACIT-F, Functional Assessment of Chronic Illness Therapy–Fatigue. PROMISE T-score Fatigue: measured by Patient Reported Outcome Measurement Information System Fatigue-Short Form (PROMIS F-SF) and converted to PROMISE-T scores; ESAS, Edmonton Symptom Assessment Scale; MFSI-SF, Multidimensional Fatigue Symptom Inventory-Short Form; FACT-G, Functional Assessment of Cancer Therapy-General; HADS, Hospital Anxiety Depression Scale.

a Determined by using the χ² test, or the Wilcoxon rank-sum test. *P* values lower than .05 were considered statistically significant.

Table 2 shows the FACIT-F scores significantly improved in both arms with mean (SD) change, effect size (ES) of 11(14), 0.87(*P <* .001); and 9(12), 0.74(*P =* .04) in MP + PA, and Pl + PA arms, respectively. We also found significant improvements in PROMIS-F, ES −1.05 (*P =* .003), MFSI-SF global, −1.32(*P <* .001), MFSI-SF general, −1.53(*P <* .001), MFSI-SF physical, −0.72(*P =* .02), MFSI-SF emotional, –0.96 (*P =* .003), MFSI-SF mental, −0.61 (*P =* .04), and HADS-depression, ES −0.92 (*P =* .004) in the combination therapy arm.

**Table 2.** Changes in cancer related fatigue and related symptoms in methylphenidate and physical activity, and placebo and physical activity arms

	MP + PA (*n* = 23)					Pl + PA (*n* = 11)				
Main outcomes, mean (SD)	Baseline	Week 2	Change	Effect size^a^	*P*^a^	Baseline	Week 2	Changes	Effect size^a^	*P*^a^
FACIT fatigue subscale	19(8)	30 (12)	11(14)	**0.87**	**<.001**	20(7)	29(11)	9(12)	**0.74**	**.04**
PROMISE T-score										
Fatigue	60(8)	52(7)	−9(9)	**−1.05**	**.003**	61(5)	55(4)	−8(7)	−1.09	.07
MFSI-SF										
Global	29(16)	17(13)	−19(14)	**−1.32**	**<.001**	32(15)	17(5)	−21(13)	**−1.61**	**.049**
General fatigue	17(5)	9(5)	−10(6)	**−1.53**	**<.001**	19(4)	13(5)	−8(6)	−1.42	.07
Physical fatigue	10(5)	7(5)	−4(5)	**−0.72**	**.02**	9(4)	5(4)	−4(4)	−0.86	.13
Emotional fatigue	8(6)	5(4)	−5(5)	**−0.96**	**.003**	7(5)	4(2)	−2(4)	−0.51	.32
Mental fatigue	8(6)	7(4)	−3(5)	**−0.61**	**.04**	9(7)	6(3)	−3(7)	−0.44	.38
Vigor	13(3)	10(4)	−2(5)	−0.52	.07	12(3)	12(2)	0(4)	−0.09	.85
FACT-G total	64(8)	58(8)	−6(10)	**−0.97**^b^	**.002**^b^	59(7)	57(17)	−7(10)	−0.65	.22
Physical well-being	12(6)	7(5)	−6(7)	**−0.92**	**.004**	8(5)	7(4)	−1(4)	−0.35	.48
Social/family well-being	25(4)	23(6)	0(4)	0.07	.79	25(4)	22(9)	−3(6)	−0.41	.41
Emotional well-being	8(4)	7(4)	0(3)	−0.62^b^	.09^b^	7(5)	7(6)	−2(3)	−0.70	.20
Functional well-being	20(5)	20(7)	1(7)	0.07	.80	19(4)	21(4)	−1(5)	−0.16	.74
HADS										
Anxiety	5(4)	5(4)	−1(3)	−0.39	.17	5(4)	3(3)	−2(2)	−0.77	.16
Depression	7(4)	5(4)	−2(3)	**−0.92**	**.004**	6(2)	5(3)	−1(1)	−0.96	.10
ESAS										
Fatigue	6(2)	4(3)	−1(3)	−0.45	.08	6(2)	5(2)	−2(3)	−0.60	.14
Pain	3(3)	2(2)	−1(3)	−0.27	.27	2(1)	2(2)	1(1)	0.48	.22

Abbreviations: MP + PA, Methylphenidate and Physical Activity arm; PL + PA, Placebo and Physical Activity arm; IQR, interquartile. FACIT-F, Functional Assessment of Chronic Illness Therapy–Fatigue, mild fatigue: 35–52. PROMISE T-Score Fatigue: measured by Patient Reported Outcome Measurement Information System Fatigue-Short Form (PROMIS F-SF) and converted to PROMISE-T scores, higher scores indicating greater fatigue, including normal limits: <55, mild: 55–60, moderate: 60–70, severe: >80. ESAS, Edmonton Symptom Assessment Scale. MFSI-SF, Multidimensional Fatigue Symptom Inventory-Short Form. FACT-G, Functional Assessment of Cancer Therapy-General, mild fatigue for FACT-G total score: 80–108, mild fatigue for Physical Well-being: 26–28, mild fatigue for Social/Family Well-being: 20–28, mild fatigue for Emotional Well-being: 22–28; mild fatigue for Functional Well-being: 21–28. HADS, Hospital Anxiety Depression Scale, mild anxiety or depression: 0–7.

a Effect sizes and *P*-values were generated and determined using different methods according to variable distributions.

b Wilcoxon signed-rank test was used for the nonparametrically distributed variables, while the paired-sample T-test was used to calculate effect sizes (Cohen’s D) and *P*-values for parametrically distributed variables.

Note: *P*-values lower than .05 were considered statistically significant. Bold effect sizes and *P*-values indicated that there were statistically significant differences between the two groups.

Table 3 summarizes the adverse events in the MP + PA and Pl + PA as assessed by NCI CTCAE version 4. No statistically significant difference in the adverse events between MP + PA and Pl + PA arms was found, *P =* .54.

**Table 3.** Summary of types of adverse events in the methylphenidate and physical activity, and placebo and physical activity arms

Adverse events (Grade: 1−2)^a^^,^^b^	MP + PA arm (*n* = 21)	Pl + PA arm (*n* = 1)	Total (*n* = 22)^c^
Abdominal pain	1	0	1
Allergic reaction	1	0	1
Anxiety	2	0	2
Atrial fibrillation	1	0	1
Blurred vision	1	0	1
Chills	1	0	1
Diarrhea	1	0	1
Dizziness	2	0	2
Dry mouth	2	0	2
Fatigue	1	0	1
Localized edema	1	0	1
Mucosal infection	0	1	1
Nausea	1	0	1
Pain	2	0	2
Pain in the extremity	2	0	2
Palpitations	1	0	1
Pancreatitis	1	0	1

Abbreviations: MP + PA arm, Methylphenidate and Physical Activity; Pl + PA arm, Placebo and Physical Activity.

In the MP + PA arm, three adverse events (allergic reaction, anxiety, and palpitations) were definitely attributed to the study treatment and two adverse events (atrial fibrillation and dizziness) were possibly attributed to the study treatment.

a Adverse events were assessed using Common Terminology Criteria for Adverse Events (CTCAE) version 4.

b No adverse events with grades higher than 2.

c No statistically significant difference of reported adverse events was detected between combination therapy and placebo groups determined by using the Fisher’s Exact Test (=.54).

## Discussion

Our pilot study is the first to evaluate the effects of MP combined with PA intervention for treating CRF in patients with advanced cancer receiving immunotherapy. CRF is highly prevalent in this population and contributes to reduced quality of life and challenges in continuing cancer treatment (Azeem Khan et al. 2021; Kiss et al. 2022). We observed significant improvements in fatigue, as measured by the FACIT-F scale, in both the MP + PA and placebo + PA groups, with no significant differences in adverse events. Notably, MP + PA led to significant improvements across multiple fatigue dimensions – physical, emotional, mental, general, and global – as assessed by the MFSI-SF, as well as depression scores (HADS).

Treatment with MP combined with PA led to improvement in CRF (Cella et al. 2002). The improvement in FACIT-F scores exceeded the 3.5-point threshold for clinical significance (Cella et al. 2002), suggesting that MP + PA may potentially offer greater benefit than PA alone (Cheville et al. 2013; Ligibel et al. 2022; Tanriverdi et al. 2023; Hiensch et al. 2024). These findings align with prior studies supporting combination approaches for CRF using PA in advanced cancer (Yennurajalingam et al. 2021, 2025). Further adequately powered studies are needed to confirm the effectiveness of MP + PA and to determine whether the combination provides a synergistic advantage over either intervention alone.

In addition to the primary outcome, our study revealed several noteworthy findings:(a) Symptom assessments after the study treatment indicated no worsening of anxiety or sleep disturbances. Combined with the recorded adverse events, these results support the safety of the combined intervention of PA with MP. These findings are consistent with findings of studies by our team and others using MP and PA independently (Bruera et al. 2006; Cheville et al. 2013; Belloni et al. 2021; Bower et al. 2024; Hiensch et al. 2024; Stone et al. 2024). (b) Patients experiencing emotional fatigue, low physical well-being, and depressive symptoms appeared to benefit particularly from the intervention. Similar findings were found in prior MP as well as PA studies (Bruera et al. 2013; Calderon et al. 2024; Hiensch et al. 2024; Stone et al. 2024). Although this may represent a trend due to the limited sample size, it remains a compelling observation that warrants further investigation in larger, adequately powered studies. (c) Consistent with prior research (Bruera et al. 2006; Stone et al. 2024), patients in the placebo arm demonstrated significant improvement in CRF and related symptoms, with an ES of 0.74. These findings underscore the importance of adequately powering future studies to account for the placebo effect.

The clinically meaningful improvements observed and the favorable safety profile of the intervention support its potential value. These findings provide a strong rationale for conducting well-powered phase III trials. Future studies should utilize a randomized, double-blind, placebo-controlled design to determine whether combining PA – or its attention control – with either MP or placebo offers superior efficacy in managing CRF in patients with advanced cancer. Our study used a patient-controlled MP dosing with a median MP dose of 10 mg of methylphenidate per day. Future studies should also investigate whether the dosing (10, 5, and 10 mg three times daily) and modality of methylphenidate administration, such as fixed versus patient-controlled dosing, may affect CRF improvement.

### Limitations

The results of this pilot study should be interpreted cautiously due to (a) the small sample size. (b) The accrual of the study was affected due to the pandemic, and the study was discontinued prior to attaining the required sample size. (c) Due to the constraints imposed by the COVID-19 pandemic, we were unable to systematically assess participant adherence to the standardized PA intervention. This limitation may have influenced the interpretation of the intervention’s effects and should be considered when evaluating the study outcomes in future studies. (d) Our study was conducted between May 2018 and August 2021. However, recent publications support the continued relevance of our findings, particularly regarding the combination of MP with PA for the treatment of CRF in patients with advanced cancer (Azeem Khan et al. 2021; Bower et al. 2024; Chin-Yee et al. 2024). These emerging studies reinforce the applicability and potential impact of our intervention in current clinical contexts.

## Conclusion

Our preliminary study found MP + PA was associated with significant improvement in CRF scores. The fatigue dimensions and depression scores significantly improved in the MP + PA arm. Further comparative studies using MP + PA for CRF are justified.
